# Mycobacterium tuberculosis Beijing genotype strains associated with febrile response to treatment.

**DOI:** 10.3201/eid0705.017518

**Published:** 2001

**Authors:** R van Crevel, R H Nelwan, W de Lenne, Y Veeraragu, A G van der Zanden, Z Amin, J W van der Meer, D van Soolingen

**Affiliations:** University Medical Center Nijmegen, The Netherlands. r.vancrevel@aig.azn.nl

## Abstract

DNA fingerprinting has demonstrated predominance of the Beijing genotype among Mycobacterium tuberculosis strains isolated in Southeast Asia. We prospectively examined the occurrence of Beijing genotype strains in tuberculosis patients in Indonesia. Early in treatment, patients infected with Beijing genotype strains more often had fever unrelated to disease severity, toxicity, or drug resistance, indicating that Beijing genotype strains may have specific pathogenic properties.


### *Mycobacterium tuberculosis* Beijing Genotype Strains Associated with Febrile Response to Treatment

**Reinout van Crevel,* Ron H.H. Nelwan,† Wilma de Lenne,* Yelilsan Veeraragu,‡ Adri G. van der Zanden,§ Zulkifli Amin,† Jos W.M. van der Meer,* and Dick van Soolingen¶**

*University Medical Center Nijmegen, Nijmegen, the Netherlands; †University of Indonesia, Jakarta, Indonesia; ‡Trisakti University, Jakarta, Indonesia; §Gelre Hospitals, Apeldoorn, the Netherlands; and ¶National Institute of Public Health and the Environment (RIVM), Bilthoven, the Netherlands

DNA fingerprinting has demonstrated predominance of the Beijing genotype among *Mycobacterium tuberculosis* strains isolated in Southeast Asia. We prospectively examined the occurrence of Beijing genotype strains in tuberculosis patients in Indonesia. Early in treatment, patients infected with Beijing genotype strains more often had fever unrelated to disease severity, toxicity, or drug resistance, indicating that Beijing genotype strains may have specific pathogenic properties.

In 1995, DNA fingerprinting showed that >80% of a collection of *Mycobacterium tuberculosis* isolates from China belonged to a genetically closely related group of bacteria, the Beijing genotype family (1). Strains of this genotype family also dominated in neighboring countries in Southeast Asia, whereas prevalence was lower on other continents (1). In a recent study, 50% of 563 isolates from Vietnam belonged to the Beijing genotype; moreover, the occurrence of these strains correlated strongly with young age and hence with active transmission of tuberculosis (TB) (2). Strains of the Beijing family were thought to have expanded recently from an evolutionary lineage with an unknown selective advantage over other *M. tuberculosis* genotypes (1). Strain W, a highly drug-resistant strain that caused large nosocomial outbreaks in New York City in the early 1990s (3,4), is an evolutionary branch of the Beijing genotype family (5).

Worldwide, Indonesia has the third highest number of TB patients, with an estimated 591,000 cases in 1998 (6). No data have been published from Indonesia on the distribution of *M. tuberculosis* genotypes.

### The Study

We prospectively collected demographic and clinical data and performed DNA fingerprinting of *M. tuberculosis* isolates from a cohort of patients in Jakarta. We assessed prevalence of the Beijing genotype strains and compared drug resistance and clinical course of patients infected with Beijing and other genotype strains of *M. tuberculosis*.

From December 1998 through March 1999, 121 consecutive patients were enrolled at Perkumpulan Pembertasan Tuberkulosa Indonesia, an outpatient TB clinic in a densely populated area in Jakarta. Informed consent was obtained from all patients. Demographic data, symptoms and signs, risk factors for TB, and details about previous antituberculosis therapy were recorded. From three large-volume sputum samples, microscopy for acid-fast bacilli (AFB) and culture for *M. tuberculosis* were performed. Chest X-rays taken before start of treatment were evaluated by two experienced pulmonologists. HIV status was determined. When TB was bacteriologically confirmed, a standard four-drug regimen (isoniazid [INH], rifampicin, pyrazinamide, and ethambutol) was prescribed (7). During follow-up examinations, symptoms, body temperature, and weight were recorded.

After standard processing of sputum samples, culture was performed in 3% Ogawa's medium. Twice a week, slants were examined for colonies. At the National Institute of Public Health and the Environment (RIVM), Bilthoven, the Netherlands, susceptibility testing of patient isolates was done by using serial dilutions of five antituberculosis drugs on Middlebrook's medium (8). For pyrazinamide, the same method was used after pH of the medium was adjusted to 5.70–5.75. MICs used to define drug resistance were INH 0.2 mg/L, rifampicin 1 mg/L, ethambutol 5 mg/L, streptomycin 5 mg/L, and pyrazinamide 50 mg/L.

Genotyping of mycobacterial isolates was done by restriction fragment length polymorphism (RFLP) typing. Extraction of DNA from *M. tuberculosis* strains and Southern blotting with labeled IS*6110* DNA as a probe were done by a standard DNA fingerprinting method (9). Spacer oligonucleotide typing (spoligotyping) of *M. tuberculosis* DNA from patient isolates was done as described (10). This method reliably identifies Beijing genotype strains on the basis of specific reaction with only 9 (35 to 43) of the 43 spacers used in spoligotyping (1,2). All the identified Beijing genotype strains also had highly similar but in most cases not identical IS*6110* RFLP patterns, with a high number of bands. From culture-negative patients, *M. tuberculosis* DNA for spoligotyping was directly isolated from sputum smears (11). Computer-assisted analysis of IS*6110* fingerprints and

Address for correspondence: Reinout van Crevel, Department of Internal Medicine, University Medical Center Nijmegen, P.O Box 9101, 6500 HB Nijmegen, the Netherlands; fax: 0031-243541734; e-mail: r.vancrevel@aig.azn.nl spoligotyping patterns was done with Bionumerics 4.0 (Applied Maths, Kortrijk, Belgium).

Descriptive result sare reported as median (range) for continuous variables and as percentages for categorical data. The Mann-Whitney U test was used for comparison of continuous variables and the Pearson chi-square test for proportions. The level of significance was $p<0.05$.

In 121 consecutive patients with a clinical diagnosis of TB, direct examination of at least one sputum smear was positive for AFB in 89 (73%), and *M. tuberculosis* was cultured from 83 (73%) of 113 cultures performed. IS*6110* restriction fragment patterns were analyzed in combination with the respective spoligopatterns of these samples (Figure). Most (90%) strains had a fingerprint pattern that differed from the RFLP patterns of other strains in this study.

Four miniclusters of two identical RFLP patterns were found. Besides visiting the same clinic, no epidemiologic connection could be made between patients infected with these strains. Laboratory contamination seems unlikely, as patient specimens were collected on separate days. DNA of two isolates did not show hybridization with the IS*6110* probe, but yielded positive reactions in spoligotyping. Such *M. tuberculosis* strains without IS*6110* DNA have been described (12). Twenty-seven strains had a high number of IS*6110*-containing restriction fragments and a high degree of similarity (≥66%) among patterns. This homogeneous group of isolates represented the Beijing family of genotypes, as confirmed by spoligotyping (Figure). Direct spoligotyping on sputum smears of culture-negative patients added another four patients with Beijing strain infections. Of 92 *M. tuberculosis* strains analyzed (83 cultured isolates by IS*6110* RFLP and spoligotyping and 9 stained sputum smears by spoligotyping only), 31 strains (34%) were Beijing genotypes.

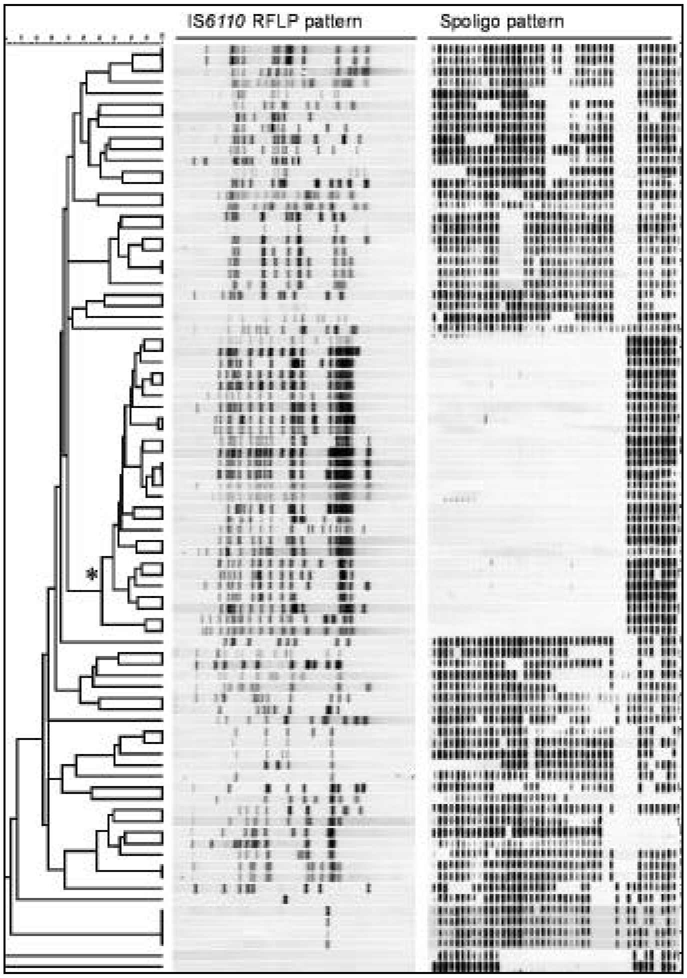

Figure. Dendrogram showing similarity of the 83 IS*6110* restriction fragment length polymorphism patterns of *Mycobacterium tuberculosis* isolates from Jakarta, in combination with the respective spoligotype patterns. The branch in the dendrogram representing Beijing genotype isolates is indicated with an asterisk (*).

Cultures became positive for *M. tuberculosis* after 4.7 weeks for Beijing strains, compared with 5.2 weeks for other strains. As in a recent study in Vietnam, drug susceptibility testing showed a trend toward higher prevalence of resistance to INH (37% vs. 20%; p=0.09) and streptomycin (15% vs. 5%; p=0.16) in Beijing strains compared with other strains; however, these differences did not attain statistical significance. (Table). The two groups did not differ significantly in prevalence of multidrug resistance (7% vs. 4%). For both Beijing and other strains, drug resistance was found equally among age groups (data not shown).

The characteristics of patients infected with Beijing genotypes (n=31) were compared with those of patients infected with other strains (n=61) (Table). The age distribution in the two groups was similar, although no patients <16 years of age were evaluated. No relation was found between genotype and BCG vaccination status (Table). Of the patients infected with Beijing strains, 7 (23%) had received one or more antituberculosis drugs before, compared with 18 (29%) of patients infected with other strains. Only two patients (one in each group) had been prescribed a standard four-drug regimen for 6 months. On entering the study, patients did not differ significantly in the presence of fever, dyspnea, or hemoptysis, or in duration of symptoms (data not shown). The nutritional status in both groups of patients was similar, as judged by body mass index (17 vs. 16.9 kg/$m^2$). The number of smear-positive patients and the AFB density in sputum smears were similar in both groups (data not shown). Patients did not differ significantly by X-ray evaluation; the number of lung fields involved was similar, and an equal percentage in both groups had pulmonary cavities (Table). Only one patient, whose initial diagnosis was cavitary TB, was HIV positive; this patient was infected with a Beijing strain.

Patients were evaluated weekly. Most patients had an early, beneficial response to treatment. In both groups, 20% continued to lose weight during the first 2 months of the study. This loss was small (approximately 1 kg) and may have been due to observance of the Ramadan fast. Body weight increased a median of 2 kg (range 0 to 8 kg) in the remaining patients in both groups. No relation was found between drug resistance and changes in body weight. At 6 months, patients in both groups had gained weight equally (median 5 kg). No patient had active disease at this point, but treatment was extended in patients infected with strains resistant to multiple drugs.

Thirty-two percent of patients developed fever (>38°C, maximum 39.3°C) during the first weeks of treatment. No patient reported shaking chills during this period. This transient febrile response, which lasted 2 to 3 weeks, was found in 15 (48%) of 31 patients infected with Beijing strains compared with 13 (21%) of 61 patients with other strains (relative risk 2.3; 95% confidence interval 1.1–4.7). Drug resistance could not account for this finding: 46% of

Table. Drug resistance and patient characteristics by *Mycobacterium tuberculosis* genotype, Indonesia

| | Genotype | |
|---|---|---|
| Patient characteristics | Beijing, n=31 (%) | Other, n=61(%) |
| Male | 19 (61) | 35 (57) |
| Age in years, median (range) | 31 (19-68) | 29 (17-70) |
| BCG scar | 8 (26) | 14 (23) |
| Chest X-ray | | |
| cavities | 16 (52) | 29/55[a] (53) |
| bilateral disease | 24 (77) | 40/55 (73) |
| Febrile response to treatment[b] | 15 (48) | 13 (21) |
| Patient strain resistant to[c] | n = 27 (%) | n = 56 (%) |
| Isoniazid | 10 (37) | 11 (20) |
| Rifampicin | 2 (7) | 2 (4) |
| Pyrazinamide | 0 | 3 (5) |
| Ethambutol | 1 (4) | 3 (5) |
| Streptomycin | 4 (15) | 3 (5) |
| Any prescribed drug | 11 (39) | 14 (25) |

[a]Six chest X-rays in the non-Beijing group were evaluated by only one pulmonologist.
[b]Significant difference between groups (Mann-Whitney U-test; p=0.02).
[c] Eighty-three isolates were available for drug susceptibility testing.

patients infected with a fully susceptible Beijing strain compared with 19% of patients infected with susceptible non-Beijing strains had a febrile response (p=0.06). Neither did severity of disease account for the difference, as the febrile response was not associated with nutritional status or presence of pulmonary cavities (data not shown). Drug toxicity, which may also induce fever, was not established.

### Conclusions

Worldwide, DNA fingerprinting has revealed extensive heterogeneity of *M. tuberculosis* genotypes (13). However, a distinct and predominant *M. tuberculosis* genotype, termed Beijing, has been found in the People's Republic of China and neighboring countries (1,14). Our study demonstrates that Beijing strains are also present in the Indonesian archipelago.

The prevalence of Beijing strains in China (85%), Mongolia (50%), South Korea (43%), Thailand (37%), Vietnam (50%), and Indonesia (34%) suggests that this clone spreads in Southeast Asia, where TB is endemic. Beijing genotype strains also account for a substantial proportion of multidrug-resistant TB cases in Azerbaijan and Estonia (15,16) and Cuba (17). Strain W, which also belongs to the Beijing genotype family (5), caused major outbreaks of multidrug-resistant TB during the past decade in the United States (3,4) and South Africa (18). In summary, all reports on the occurrence of Beijing genotypes show a clear correlation with drug resistance. In our study, 37% of the Beijing strains were resistant to INH. However, multidrug resistance was limited, making drug resistance unlikely as a single explanation for the predominance of Beijing strains in this population.

Different transmission rates may account for an unequal distribution of genotypes. In a TB outbreak in the United States, a particular *M. tuberculosis* genotype caused extensive transmission, as evaluated by skin test conversion (19). In Indonesia, outbreak investigations like these seem impossible in light of the high prevalence of TB and standard BCG vaccination, which hampers interpretation of tuberculin skin tests. However, indirect evidence supporting increased transmission of Beijing strains comes from a recent study in Vietnam, which demonstrated that Beijing strains were more prevalent among young patients (2). We could not confirm this in the Indonesian patients, but we did not investigate patients <16 years of age. In agreement with the study in Vietnam, we found no correlation between vaccination status and genotypes.

Disease severity in patients infected with Beijing or other genotypes seemed similar. However, our prospective evaluation revealed a different response to treatment. Forty-eight percent of the patients infected with Beijing strains and 21% of the patients infected with other strains had a transient febrile response shortly after start of treatment. Disease severity, drug toxicity, and drug resistance could not account for this difference. The increased risk of febrile response in patients infected with Beijing strains is unusual and suggests that these strains induce a different host response. In support of this hypothesis, Beijing genotype strains were recently found to elicit an altered cytokine response in two animal models (D. van Soolingen, unpub. data). This differential response may be related to the rapid spread of Beijing genotype strains. There may be an interesting parallel with the outbreak in the United States (19), since the causative strain in that outbreak, designated CDC1551, induced a more rapid and robust in-vitro production of pyrogenic cytokines such as interleukin-6 and tumor necrosis factor-$\alpha$ (20). Whether Beijing strains also elicit a different cytokine response in TB patients is a subject for future study.

### Acknowledgment

We thank staff members of the outpatient clinic of Perkumpulan Pembertasan Tuberkulosa Indonesia in Central Jakarta for their help, Dr. Judanarso Dawud for providing laboratory facilities, and especially Dr. Julianti Gunawan of the Microbiology Department of Persahabatan Hospital. Dr. Iskandar Zulkarnain, head of the Division of Tropical Medicine and Infectious Diseases, University of Indonesia, provided staff to conduct this study. In the Netherlands, Jan Henraat and Mirjam Dessens performed drug susceptibility testing of patient isolates, and Petra de Haas conducted DNA fingerprinting and helped with data analysis.

Reinout van Crevel is supported by the Dutch Organization for Scientific Research NWO (SGO Stipendium Infectious Diseases). Further financial support for this study was obtained from KLM Royal Dutch Airlines, the Royal Netherlands Tuberculosis Association, and the Van Deventer-Maas Stichting.

Dr. van Crevel is a resident in internal medicine at the University Medical Center Nijmegen, the Netherlands. His main research interest is the role of host defense in TB. In addition he is involved in clinical, bacteriologic, and operational studies on tuberculosis in Indonesia.

### References

1. van Soolingen D, Qian L, de Haas PE, Douglas JT, Traore H, Portaels F, et al. Predominance of a single genotype of *Mycobacterium tuberculosis* in countries of east Asia. J Clin Microbiol 1995;33:3234-8.
2. Anh DD, Borgdorff MW, Van LN, Lan NTN, van Gorkum T, Kremer K, et al. 'Beijing' genotype emerging in Vietnam. Emerg Infect Dis 2000;6:302-5.
3. Agerton TB, Valway SE, Blinkhorn RJ, Shilkret KL, Reves R, Schluter WW, et al. Spread of strain W, a highly drug-resistant strain of *Mycobacterium tuberculosis*, across the United States. Clin Infect Dis 1999;29:85-92.
4. Frieden TR, Sherman LF, Maw KL, Fujiwara PI, Crawford JT, Nivin B, et al. A multi-institutional outbreak of highly drug-resistant tuberculosis: epidemiology and clinical outcomes. JAMA 1996;276:1229-35.
5. Kurepina NE, Sreevatsan S, Plikaytis BB, Bifan PJ, Connell ND, Donnelly RJ, et al. Characterization of the phylogenetic distribution and chromosomal insertion sites of five IS*6110* elements in *Mycobacterium tuberculosis*: non-random integration in the *dnaA* -*dnaN* region. Tuber Lung Dis 1998;79:31-42.
6. World Health Organization. Global tuberculosis control: 2000. Geneva, Switzerland, 2000.
7. World Health Organization. Treatment of tuberculosis: Guidelines for national programmes. Geneva: The Organization; 1996.
8. Gangadharam PR. Drug resistance in Mycobacteria. CRC Press, Boca Raton (FL): CRC Press; 1984.
9. Van Embden JD, Cave MD, Crawford JT, Dale JW, Eisenach KD, Gicquel B, et al. Strain identification of *Mycobacterium tuberculosis* by DNA fingerprinting: recommendations for a standardized methodology. J Clin Microbiol 1993;31:406-9.
10. Kamerbeek J, Schouls L, Kolk A, van Agterveld M, van Soolingen D, Kuijper S, et al. Simultaneous detection and strain differentiation of *Mycobacterium tuberculosis* for diagnosis and epidemiology. J Clin Microbiol 1997;35:907-14.
11. van der Zanden AG, Hoentjen AH, Heilmann FG, Weltevreden EF, Schouls LM, Van Embden JD. Simultaneous detection and strain differentiation of *Mycobacterium tuberculosis* complex in paraffin wax embedded tissues and in stained microscopic preparations. Mol Pathol 1998;51:209-14.
12. van Soolingen D, de Haas PE, Hermans PW, Groenen PM, Van Embden DE. Comparison of various repetitive DNA elements as genetic markers for strain differentiation and epidemiology of *Mycobacterium tuberculosis*. J Clin Microbiol 1993;31:1987-95.
13. van Soolingen D, Hermans PW, de Haas PE, Soll DR, Van Embden JD. Occurrence and stability of insertion sequences in *Mycobacterium tuberculosis* complex strains: evaluation of an insertion sequence-dependent DNA polymorphism as a tool in the epidemiology of tuberculosis. J Clin Microbiol 1991;29:2578-86.
14. Park YK, Bai GH, Kim SJ. Restriction fragment length polymorphism analysis of *Mycobacterium tuberculosis* isolated from countries in the western Pacific region. J Clin Microbiol 2000;38:191-7.
15. Marttila HJ, Soini H, Eerola E, Vyshnevskaya E, Vyshneskiy BI, Otten TF, et al. A Ser315Thr substitution in KatG is predominant in genetically heterogeneous multidrug-resistant *Mycobacterium tuberculosis* isolates originating from the St. Petersburg area in Russia. Antimicrob Agents Chemother 1998;42:2443-5.
16. Niemann S, Rusch Gerdes S, Richter E. IS6110 fingerprinting of drug-resistant *Mycobacterium tuberculosis* strains isolated in Germany during 1995. J Clin Microbiol 1997;35:3015-20.
17. Diaz R, Kremer K, de Haas PE, Gomez RI, Marrero A, Valdivia JA, et al. Molecular epidemiology of tuberculosis in Cuba outside of Havana, July 1994-June 1995: utility of spoligotyping versus IS6110 restriction fragment length polymorphism. Int J Tuberc Lung Dis 1998;2:743-50.
18. van Rie A, Warren RM, Beyers N, Gie RP, Classen CN, Richardson M, et al. Transmission of a multidrug-resistant *Mycobacterium tuberculosis* strain resembling “strain W” among noninstitutionalized, human immunodeficiency virus-seronegative patients. J Infect Dis 1999;180:1608-15.
19. Valway SE, Sanchez MP, Shinnick TF, Orme I, Agerton T, Hoy D, et al. An outbreak involving extensive transmission of a virulent strain of *Mycobacterium tuberculosis*. N Engl J Med 1998;338:633-9.
20. Manca C, Tsenova L, Barry CE, Bergtold A, Freeman S, Haslett PA, et al. *Mycobacterium tuberculosis* CDC1551 induces a more vigorous host response in vivo and in vitro, but is not more virulent than other clinical isolates. J Immunol 1999;162:6740-6.
